# Detection of Soluble Solid Content in Citrus Fruits Using Hyperspectral Imaging with Machine and Deep Learning: A Comparative Study of Two Citrus Cultivars

**DOI:** 10.3390/foods14122091

**Published:** 2025-06-13

**Authors:** Yuxin Xiao, Yuanning Zhai, Lei Zhou, Yiming Yin, Hengnian Qi, Chu Zhang

**Affiliations:** 1 School of Information Engineering, Huzhou University, Huzhou 313000, China; 2 College of Mechanical and Electronic Engineering, Nanjing Forestry University, Nanjing 210037, China; 3 Economic Crop Technology Promotion Station, Huzhou Academy of Agricultural Sciences, Huzhou 313000, China

**Keywords:** hyperspectral imaging, citrus quality, machine learning, deep learning, SHAP

## Abstract

Hyperspectral imaging (HSI) has broad applications for detecting the soluble solids content (SSC) of fruits. This study explores the integration of HSI with machine learning and deep learning to predict SSC in two mandarin varieties: Ponkan and Tianchao. Traditional machine learning models (support vector machines and partial least squares regression) and deep learning models (convolutional neural networks, long short-term memory, and Transformer architectures) were evaluated for SSC prediction performance. Combined models that integrated different deep learning architectures were also explored. Results revealed varietal differences in prediction performance. For Ponkan mandarins, the best SSC prediction model was achieved by partial least squares regression, outperforming deep learning models. In contrast, for Tianchao mandarins, the deep learning model based on convolutional neural network slightly surpassed the traditional model. SHapley Additive exPlanations (SHAP) analysis indicated that the influential wavelengths varied between varieties, suggesting differences in key spectral features for SSC prediction. These findings highlight the potential of combining HSI with advanced modeling for citrus SSC prediction, while emphasizing the need for variety-specific models. Future research should focus on developing more robust and generalized prediction models by incorporating a broader range of citrus varieties and exploring the impact of varietal characteristics on model performance.

## 1. Introduction

Citrus is one of the most popular fruits in the world, being planted in over 140 countries. Citrus is a family of fruits with unique nutrients (vitamins, minerals, and dietary fiber), tastes, and aromas. Soluble solid content (SSC) is one of the main quality attributes of citrus fruits, being the focus of attention among planters, consumers, and traders. As a key indicator of sweetness and ripeness, SSC plays a vital role in determining fruit marketability and consumer preference. Reliable and accurate measurement of SSC is therefore essential for optimizing harvest timing, post-harvest handling, and quality control. The general approach to measuring the SSC of the citrus is to use a digital refractometer. This method is destructive, as the fruit must be squeezed into juice for measurement. Thus, this method is generally used for sampling, and measuring the SSC of all fruits using a digital refractometer is quite difficult. Developing the non-destructive technologies for rapid and accurate prediction of SSC of citrus fruits is of great importance.

Visible/near-infrared spectroscopy (VNIRS) and hyperspectral imaging (HSI) technology have shown great promise for the non-destructive and rapid assessment of fruit quality attributes, including the SSC [1]. Theoretically, the spectral profiles reflect the information of the overtones and combinations of vibrational modes of chemical bonds. These chemical bonds can be found in the chemical components of fruits, including the chemical components of SSC. Thus, the spectral profiles obtained from VNIRS and HSI can be used to measure the SSC in fruits. However, VNIRS generally conducts point-scan, lacking spatial information. In addition to VNIRS, hyperspectral imaging, which integrates spatial and spectral information simultaneously, can obtain spectral information from the entire sampling region, providing more comprehensive information. Thus, the spectral information can be obtained from the entire fruit to represent the sample using hyperspectral imaging, reducing variations within the sample. This characteristic makes hyperspectral imaging a reliable analytical technology for fruit quality and safety inspection, including SSC prediction. Although the success of the research on VNIRS and HSI in fruit quality and safety inspection has been widely reported [1], there is still a long way to developing VNIRS and HSI for real-world applications. One of the challenges is that there are various varieties of fruits. Studies have shown that varietal differences existed in SSC prediction using VNIRS and HIS [2,3,4]. Variations in physicochemical properties and spectral responses between varieties make it hard to directly apply the models established for one variety to the other varieties.

Various data analysis procedures have been explored for SSC prediction using VNIRS and HSI to pursue better and robust prediction performances. Traditional machine learning methods, such as partial least squares regression (PLSR) and support vector regression (SVR), have been widely applied for SSC prediction of citrus fruits based on the spectral data [5,6,7,8]. Deep learning approaches, such as convolutional neural networks (CNNs), long short-term memory (LSTM) networks, and Transformer architectures, have demonstrated superior capability in automatic feature extraction and nonlinear modeling, offering new possibilities for spectral data analysis. Studies have shown the success of deep learning approaches for fruit quality inspection, including the prediction of SSC [1,6,8,9,10]. Different deep learning methods can learn the features from different aspects. Thus, the combination of different deep learning algorithms has been widely used and has shown success [6,11,12]. Comparison between traditional machine learning methods and deep learning methods has also been explored, and differentiated results could be observed in different studies. In some studies, traditional machine learning algorithms outperformed the deep learning models [13,14]. While in some other studies, the deep learning models outperformed the traditional machine learning models [15,16,17,18,19,20].

In this study, hyperspectral imaging with both machine learning and deep learning methods to predict SSC in two citrus varieties (Ponkan mandarin and Tianchao mandarin) under the same experimental conditions was explored. The primary objective was to explore the varietal differences in the SSC prediction of the two varieties and to explore the differences in the performance of SSC prediction using the traditional machine learning methods and the deep learning methods. Traditional machine learning methods (PLSR and SVR), deep learning methods (CNN, LSTM, Transformer), and combined deep learning methods (CNN–LSTM, LSTM–CNN, CNN–Transformer, Transformer–CNN) were systematically explored and compared for the two varieties of citrus fruits. Additionally, SHapley Additive exPlanations (SHAP) analyses of the optimal models were used to explore the similarities and differences in the key wavelengths for SSC prediction of the two varieties of citrus fruits. The main contribution of this research was to explore the impact of the varietal differences on SSC prediction performance and various modeling methods.

## 2. Materials and Methods

### 2.1. Sample Preparation

The research subjects of the experiment were Ponkan mandarins and Tianchao mandarins from a local orchard in Quzhou, Zhejiang Province, China. A total of 288 samples of Ponkan mandarin and 280 samples of Tianchao mandarin were collected. Details about Ponkan can be found in the literature [21]. No mechanical damage was found in the fruit. All samples were stored at controlled room temperature (about 15 °C) to minimize variation in sample quality prior to measurement. Each day, about 60 fruits were used for hyperspectral spectrometer scanning and the measurement of SSC. The experiments on Ponkan mandarin and Tianchao mandarin were conducted on the same batch, and the experimental conditions of the two varieties of citrus fruits were the same. Figures S1 and S2 (in the Supplementary Materials) show the images of the two varieties of citrus fruits.

### 2.2. Hyperspectral Spectrometer and Spectra Acquisition

#### 2.2.1. Hyperspectral Data Acquisition

The hyperspectral imaging system consists of an electrically controlled sample displacement scanning stage (the LabScanner, Spectral Imaging Ltd., Oulu, Finland), a hyperspectral camera (FX10, SPECIM, Spectral Imaging Ltd., Oulu, Finland), and a dual halogen lamp for uniform illumination. The LabScanner utilizes a stepper motor for scanning control; it has a high-purity black surface and is equipped with a white calibration board. The FX10 hyperspectral camera covers a spectral range of 400–1000 nm, with a spectral resolution of 5.5 nm, and a spatial pixel count of 1024 pixels. Data acquisition was conducted using the LUMO Scanner 2020 software (Spectral Imaging Ltd., Oulu, Finland).

Undamaged citrus samples were placed on the scanning stage for hyperspectral image acquisition. Six or eight samples were scanned at a time, with all samples numbered sequentially. Prior to data collection, the HSI was powered for 30 min for warm-up. The LabScanner was set to a speed of 17 mm/s, and the exposure time was set to 13.59 ms. The hyperspectral images of the navel, pedicel, and two equatorial planes of each fruit sample were acquired. The spectral characteristics of the navel and pedicel are relatively stable and less affected by fruit size and shape. The spectral profiles of two equatorial planes would help to acquire comprehensive spectral information of the fruit. Thus, the hyperspectral images of the navel, the pedicel, and two equatorial planes were acquired, resulting in four hyperspectral images of one fruit. The acquired hyperspectral image is corrected using the black and white reference images, as shown in Equation (1):

(1) $$R=\frac{I-B}{W-B}$$

where *I* represents the raw citrus data, *B* stands for the blackboard data, *W* represents the whiteboard data, and *R* denotes the corrected citrus hyperspectral image after calibration.

#### 2.2.2. Spectra Extraction

To extract the spectra from the hyperspectral images, a masking method was used to isolate the fruits from the background. This method binarized the RGB image (which was generated by the software during the hyperspectral image acquisition) and obtained the connected domain of the target sample based on the set parameters. An image mask was created using connected domains and then applied to the hyperspectral images to separate the citrus samples from the background. The spectral information of individual citrus was extracted based on the collection order, and the entire area of each citrus served as the region of interest (ROI). Next, the average spectrum was calculated by averaging the spectra of all pixels in the ROI. Then, the spectrum of the citrus fruit was averaged using the average spectra of the four sides.

### 2.3. Measurement of Soluble Solid Content

The experimental arrangement for measuring the SSC of citrus included a WIGGENS BR0035 Digital Brix Refractometer (WIGGENS Technology Ltd., Beijing, China) with an accuracy of 0.1, and a °Brix range of 0–35%. The citrus fruit was cut into three equal pieces, and then each piece of fruit was squeezed into juice. The juices were filtered by a 60 mesh gauze, and then the juices were used to measure SSC using the digital refractometer. To avoid contamination, the squeezer, the refractometer, and the beaker were washed and dried. Thus, the SSC values of the three pieces were averaged as the SSC value of the sample.

### 2.4. Spectra Preprocessing

Different sampling positions are among the key factors affecting the accuracy and robust calibration of the model of SSC prediction. The average spectrum shows better performance than the unit set spectrum. Therefore, in this study, the average spectra of the corresponding four measurement points on each sample were averaged to represent the spectrum of the sample. During the process of spectral data collection, various types of noise (i.e., interference from the instrument itself and environmental factors) can affect the quality of the collected data. In this study, wavelengths with significant noise signals at both ends of the spectral range were not included in the data analysis. Therefore, the spectral range used in the HSI dataset was 489–976 nm, consisting of 358 wavebands.

### 2.5. Data Analysis Methods

#### 2.5.1. PLSR

PLSR [22] is a widely adopted regression analysis technique that integrates the characteristics of principal component analysis and multiple linear regression. There are clear advantages in addressing challenges such as multicollinearity among independent variables and limited sample size. PLSR is frequently utilized for dimensionality reduction and modeling in high-dimensional datasets, particularly in spectral analysis and chemometrics. As a robust analytical tool, it excels in managing multivariate datasets and exhibits superior performance in predictive modeling. The primary goal of PLSR is to maximize the covariance between independent and dependent variables while minimizing prediction errors. Additionally, PLSR effectively addresses multiple linear problems, mitigating issues related to parameter estimation instability and unreliable results caused by multicollinearity.

#### 2.5.2. SVR

SVR [23] is a regression method derived from the Support Vector Machine (SVM) theory and is widely applied in spectral data analysis. SVR transforms input features into high-dimensional spaces to locate the ideal hyperplane that accurately represents the data via kernel functions. This approach enables SVR to effectively handle both linear and nonlinear relationships. The choice of kernel function, such as the radial basis function (RBF), significantly affects model performance and is critical for capturing the underlying data structure. In this research, linear functions were used as kernel functions. The general ranges of parameter C were set to 10^−7^–10^7^. Default values were used for the other parameters. A grid-search approach with 10-fold cross-validation was used to find the optimal parameters with the optimal SVR model.

#### 2.5.3. CNNs

CNNs [24] are a class of deep learning models specifically designed to process data with a grid-like structure, such as images or spectral data. A CNN typically consists of multiple layers, including convolutional layers, pooling layers, and fully connected layers [25]. The convolutional layers apply a series of learnable filters to automatically extract hierarchical features from the input data, while pooling layers reduce the spatial dimensions, improving computational efficiency and mitigating overfitting. Fully connected layers are used for final prediction tasks based on the extracted features. In hyperspectral data analysis, a CNN can effectively capture local spectral–spatial correlations, enabling improved feature representation and robust predictive performance. Compared to traditional machine learning methods, it eliminates the need for separate steps of feature selection and dimensionality reduction. Due to their strong ability to learn from complex and high-dimensional data, CNNs have been widely adopted in various fields of agricultural product quality assessment, including the detection of SSC in fruits.

The CNN model used in this study was constructed to predict SSC based on the Ponkan and Tianchao citrus datasets. MSELoss was used as the loss function, and Adam was used as the model optimization algorithm. These models include convolutional layers, pooling layers, batchnorm layers, dropout layers, flatten layers, and fully connected layers. For the SSC prediction of Ponkan citrus, the number of input variables was 1, and there were three convolutional layers (kernel_size was 5, 3, and 3, respectively) and four fully connected layers. For the SSC prediction of Tianchao citrus, the number of input variables was 1, there were two convolutional layers (kernel_size was 3 and 3, respectively), and there were three fully connected layers. Figure S3 illustrates the CNN model based on HSI data. For the SSC prediction of the Ponkan mandarin, the learning rate was set to 0.00008. For the SSC prediction of the Tianchao mandarin, the learning rate was set to 0.001.

#### 2.5.4. LSTM

LSTM [26] is a special type of recurrent neural network (RNN), which is specially designed to solve the gradient vanishing and gradient explosion problems that traditional RNNs are prone to when processing long sequence data. LSTM can effectively capture long-term dependencies in the sequence modeling process by introducing gating mechanisms (including input, forget, and output) to control the inflow, retention, and output of information. Specifically, the LSTM maintains a separate cell state at each time step, which can selectively increase or decrease information through a gating structure, so that important information can be retained over a long period of time, while irrelevant or interfering information can be effectively forgotten. This mechanism makes LSTM excellent for tasks such as series data modeling, time series forecasting, natural language processing, and spectral data analysis. In this study, LSTM was used to model the potential temporal characteristics of wavelength sequences in hyperspectral data to improve the adaptability of the SSC prediction model to spectral changes.

In this study, the LSTM model was constructed to predict SSC based on the HSI dataset of Ponkan and Tianchao. These models include LSTM layers, hidden layers, and fully connected layers. For the SSC prediction for Ponkan, the number of input variables was 358, and there were two LSTM layers (hidden_size was 128 and 256, respectively), and two fully connected layers. For the SSC prediction of Tianchao, the number of input variables was 358, and there were two LSTM layers (hidden_size was 256 and 128, respectively), and three fully connected layers. MSELoss was used as the loss function, Adam was used as the model optimization algorithm, and the learning rate was set to 0.001. Figure S4 illustrates the LSTM model based on HSI data.

#### 2.5.5. CNN–LSTM and LSTM–CNN

CNN–LSTM combines the advantages of CNN and LSTM. Firstly, CNN extracts local spatial features from hyperspectral data through a convolution operation, which strengthens the local representation ability of the data. Subsequently, the extracted feature series are input to the LSTM, and the long-term and short-term dependencies in the time series are modeled through the gating mechanism. The fusion structure can simultaneously capture the spatial pattern and wavelength sequence changes of spectral data, which improves the model’s ability to perceive and predict SSC changes.

The LSTM–CNN takes LSTM as the front end of feature extraction, first models the temporal characteristics and long dependencies in the spectral sequence, and extracts feature representations with temporal correlation. Subsequently, the extracted features are fed into the CNN module to further mine the local spatial features through convolution operations. This structure is suitable for SSC prediction tasks that emphasize the trend of spectral variation with wavelength and require high local detail features.

In this study, CNN–LSTM and LSTM–CNN fusion models were used to predict the SSC of two different citrus varieties. CNN–LSTM includes a convolution layer, LSTM layer, pooling layer, BatchNorm layer, and fully connected layer. For the SSC prediction of Ponkan and Tianchao, the number of input variables was 1, and there was 1 convolution layer (kernel_size was 3), and two LSTM layers (hidden_size was 32 and 64, respectively). The LSTM–CNN includes an LSTM layer, convolution layer, pooling layer, BatchNorm layer, and a fully connected layer. The number of input variables was 358, and there were two LSTM layers (hidden_size was 128 and 64, respectively), with two convolutional layers (kernel_size was 3 for both). Ponkan contained two fully connected layers, and Tianchao contained three fully connected layers. With MSELoss as the loss function and Adam as the model optimization algorithm, the learning rate was set to 0.0005. Figure S5 illustrates the CNN–LSTM model and LSTM–CNN model based on HSI data.

#### 2.5.6. Transformer

Transformer [27] is a deep learning model based on a self-attention mechanism, which can handle long-distance dependencies in sequence data without recursive structures. Its core advantage is the ability to process data in parallel, reducing the computational burden of traditional RNN training. In hyperspectral data analysis, Transformer can capture global features through the self-attention mechanism and fully model the complex dependence between wavelengths. Compared with traditional convolutional networks, Transformers can better capture global nonlinear associations and are suitable for prediction tasks of complex features such as SSC.

In this study, a Transformer-based regression model was established to predict SSC in hyperspectral data. Firstly, the input projection layer was used to map the original spectral features to a high-dimensional space suitable for Transformer processing. To fuse the sequence information, based on the traditional sinusoidal coding method, a position coding module was added to the projection input. The encoded input was then passed through a Transformer encoder that consists of a stack of encoder layers. Each encoder layer contained a multi-head self-attention mechanism and a feedforward neural network. For the SSC prediction for Ponkan, the number of heads in the multi-head attention mechanism was 8, and the number of encoder layers was 2; for the SSC prediction for Tianchao, the number of heads in the multi-head attention mechanism was 4, and the number of encoder layers was 2. Finally, the fully connected layer was used to map the aggregate features to a single scalar output corresponding to the predicted SSC values. Figure S6 illustrates the Transformer model based on HSI data.

#### 2.5.7. CNN–Transformer and Transformer–CNN

CNN–Transformer first uses CNN to extract local features in hyperspectral data, such as subtle changes between continuous bands or local textures. The extracted features are fed into the Transformer structure, which is modeled globally through the self-attention mechanism to capture the complex correlation between long-distance bands. The fusion strategy gives full play to the complementary advantages of local feature extraction and global dependency modeling and significantly improves the ability to characterize complex spectral changes.

The Transformer–CNN is in the reverse order from the CNN–Transformer. The Transformer module is used to model the original spectral data globally to capture the long-distance correlation features between different bands. Subsequently, CNN was used to further extract the local spatial features to strengthen the local representation ability of the features. The fusion structure is suitable for modeling complex spectral data with strong non-local correlation, which can improve the prediction accuracy and enhance the generalization ability of the model.

The CNN–Transformer hybrid model proposed in this paper first used a CNN-based feature extractor composed of convolutional blocks. Each block consisted of convolutional layers, batch normalization, ReLU activation, and max pooling, progressively transforming the input spectral sequence into higher-level local features while reducing its length. The extracted feature maps were then rearranged to match the input format required by the Transformer encoder, and sinusoidal position encoding was added to preserve the original wavelength sequence information. Subsequently, the feature sequences were processed through multiple Transformer encoder layers, each containing a multi-head self-attention mechanism and a position feedforward network, enabling the model to capture remote interactions between different spectral regions. After Transformer encoding, the output was aggregated by an average pool across sequence dimensions to obtain a fixed-size embedding for each sample. Finally, this embedding passed through a series of fully connected layers with nonlinear activation, ultimately outputting a single scalar value representing SSC. For the SSC prediction for Ponkan, the CNN input dimension was 1, there were three convolutional layer modules (kernel_size was 3, 3, and 3, respectively), the number of heads of the Transformer’s multi-head attention mechanism was 4, and the number of encoder layers was two. For the SSC prediction for Tianchao, the CNN input dimension was 1, there were two convolutional layer modules (kernel_size was 3 and 3, respectively), the number of heads of the Transformer multi-head attention mechanism was four, and the number of encoder layers was two.

In this study, a Transformer–CNN based on the fusion of a Transformer and a CNN was proposed. Firstly, the hyperspectral data was projected into a higher-dimensional feature space using a linear transformation to enhance the representation ability. Positional encoding based on sine and cosine functions was then added to the input feature to explicitly inject sequence position information. The position-encoded features were imported into a stacked Transformer encoder. Each encoder layer consists of a multi-head attention mechanism and a feedforward neural network, and both Ponkan and Tianchao used two layers of encoders, each containing four attention heads. The Transformer encoded output was rearranged to fit the CNN input and then input into a two-layer one-dimensional convolutional neural network for local feature extraction (kernel_size was 3, 3, and 3, respectively), followed by batch normalization (BatchNorm), ReLU activation function, and maximum pooling layer. The convoluted output was flattened and passed through three fully connected layers, which are successively reduced in dimension and transformed nonlinearly (ReLU activated). Finally, the scalar is output, which represents the SSC prediction value of the corresponding sample. Figure S7 illustrates the CNN–Transformer model and the Transformer–CNN model based on HSI data.

### 2.6. Software and Model Evaluation Metrics

#### 2.6.1. SHAP Analysis

SHAP analysis [28], rooted in game theory, was utilized to quantify the contribution (SHAP value) of each input feature based on its average influence on model outputs, thus enabling interpretation of the optimal models. In this study, SHAP values were calculated using the entire training sets of the optimal machine learning and deep learning models, which were subsequently selected for further analysis. Summary plots were generated to visualize the magnitude and direction of spectral band influences in the training sets of both modeling approaches.

#### 2.6.2. Model Evaluation Metrics and Software Environment

In this paper, the performances of models were evaluated by root mean square error (RMSE) and correlation coefficient (*r*) of the training, validation, and test sets. A higher *r* and a lower RMSE indicate better predictive capability of the model. The models with higher *r* (close to 1) and lower RMSE values (close to 0) were considered as the better models.

**Figure 1:**
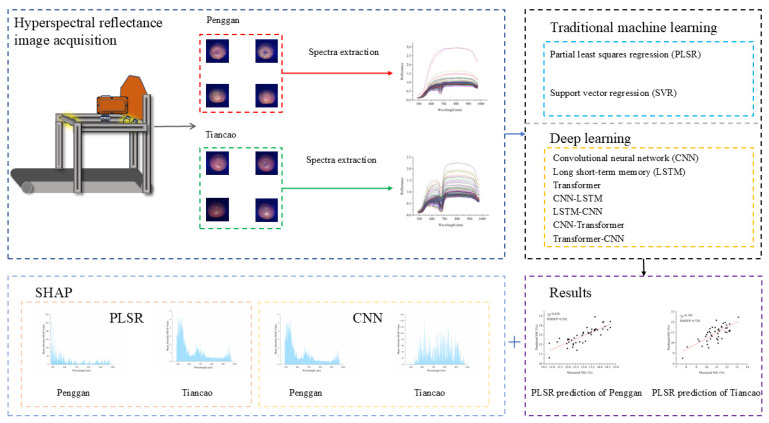
Flowchart of this research.

All the spectra extraction and modeling were performed using Python 3.9.13. PLSR and SVR were conducted using Scikit-learn 1.1.3. CNN, LSTM, Transformer, CNN–LSTM, LSTM–CNN, CNN–Transformer, and Transformer–CNN were conducted using PyTorch 1.11.0. Figure 1 shows the research flowchart of this study.

## 3. Results

### 3.1. Spectral Profiles and Outlier Removal

**Figure 2:**
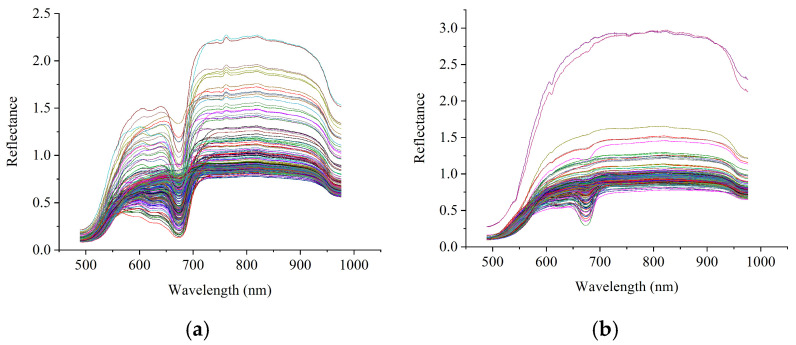
Spectral profiles of the Ponkan mandarin and Tianchao mandarin: (**a**) Ponkan mandarin; (**b**) Tianchao mandarin. The different color lines represent the spectra of different samples.

In this study, four sampling locations in the navel, pedicle, and two equatorial planes were selected, and spectral data were collected and averaged to obtain the average spectrum of each sample. Figure 2 shows the spectral data for two citrus varieties. It can be observed that the general spectral curves of the two oranges are very similar, with slight differences observed at different wavelengths. It can also be observed that some of the spectra had relatively higher reflectance, which was caused by the sample size.

In this study, a total of 288 Ponkan mandarins and 280 Tianchao mandarins were measured. During the acquisition of spectral data and the measurement of SSC, interferences and errors can occur, and outliers may occur. Anomalous samples were removed prior to further analysis [7]. The method of identifying outliers is to use all samples to build a PLSR model and make predictions to calculate the error of each sample, and finally select 12 samples with large prediction errors to be manually identified as outliers (which has a negative impact on the model). A total of 12 sample anomalies were identified in Ponkan and Tianchao and removed from the average spectral dataset. Next, the remaining samples were randomly divided into training, validation, and testing, set to a ratio of 4:1:1, and the remaining samples were put into the training set. The statistical analysis of SSC for the two types of citrus fruit is presented in Table 1. Differences could be found for the SSC distributions of Ponkan and Tianchao.

### 3.2. Prediction Results of Regression Models

#### 3.2.1. Traditional Machine Learning Models for SSC Prediction

**Figure 3:**
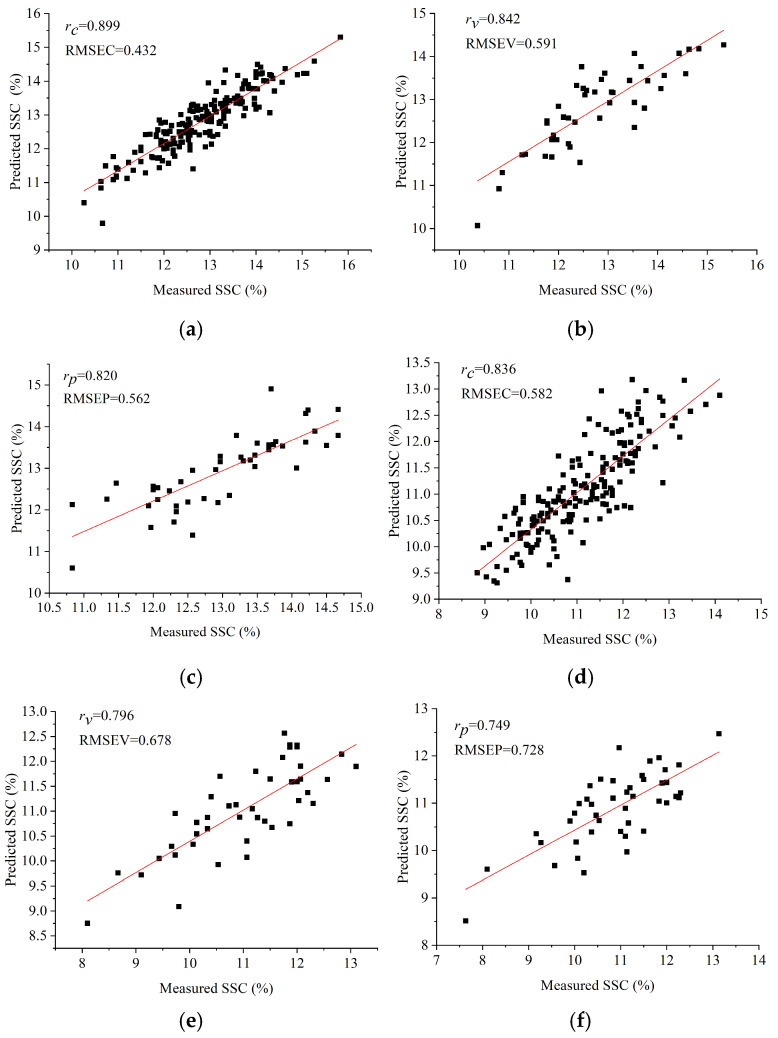
The scatter plots of measure values vs. prediction values of the PLSR models using average spectra. (**a**) training set of Ponkan; (**b**) validation set of Ponkan; (**c**) testing set of Ponkan; (**d**) training set of Tianchao; (**e**) validation set of Tianchao; (**f**) testing set of Tianchao.

Traditional machine learning models, PLSR and SVR, were established based on the divided datasets of Ponkan and Tianchao. The results are shown in Table 2. It could be observed that for Ponkan, the PLSR model obtained relatively better performance, with *r*_c_, *r*_v_, and *r*_p_ all greater than 0.8. For Tianchao, the PLSR model also outperformed the SVR model. The scatter plots of measured values vs. prediction values of the PLSR models are shown in Figure 3. As shown in Table 2, the differences in the performances of PLSR models for the two varieties of citrus fruits were much larger than those of SVR models. The reason might be that the modeling principles of PLSR and SVR were different. PLSR worked well on linear issues, and the linear relationship between the extracted spectra and the measured SSC of Ponkan might be stronger than that of Tianchao in this study. The overall prediction performances of Ponkan fruits were better than those of Tianchao fruits.

#### 3.2.2. Deep Learning Models for SSC Prediction

In addition to the traditional machine learning algorithms, deep learning-based regression models were obtained, and the results are shown in Table 3. The deep learning models included the widely used single model (CNN, LSTM, and Transformer), and the combination of these models (CNN–LSTM, LSTM–CNN, CNN–Transformer, and Transformer–CNN).

For SSC prediction of Ponkan mandarin, the CNN–LSTM model obtained optimal performances, followed by LSTM–CNN, with *r*_c_, *r*_v_, and *r*_p_ all over 0.7. These two models outperformed the other deep learning models. For the SSC prediction of Tianchao mandarins, the CNN model obtained relatively better performances. The performance of the CNN–LSTM model was second only to the CNN model. The *r*_c_, *r*_v_, and *r*_p_ of these two models were all over 0.7. From these results, it could be found that the performance of the deep learning models varied.

As shown in Table 3, the *r*_c_, *r*_v_, and *r*_p_ of all deep learning models were over 0.6. Differences could also be observed for the prediction performances of the two varieties of citrus fruits. It can be concluded from Table 3 that the overall prediction performances of Ponkan fruits using deep learning models were slightly better than those of Tianchao fruits using deep learning models.

#### 3.2.3. Comparison Between the Traditional Machine Learning and Deep Learning Models

Further comparison was made between the traditional machine learning models and the deep learning models. As for Ponkan, the PLSR model showed the best performance. SVR, CNN–LSTM, and LSTM–CNN models obtained close results. As for Tianchao, the CNN model and PLSR model obtained close results, and the CNN–LSTM model and SVR model obtained close results. As presented in Table 2 and Table 3, the results of deep learning models were not superior to those of the traditional machine learning algorithms for both varieties of citrus fruits.

### 3.3. Model Visualization

In order to understand which feature plays a leading role in the overall prediction of the model, the SHAP was introduced to interpret the model results and to provide support for the reliability of the model results. The SHAP method is not only suitable for explaining individual predictions (local interpretation) but can also be used to evaluate the importance of the entire model (global interpretation). By interpreting multiple samples, we can obtain the overall feature importance of the model, which helps us understand which features play a leading role in the overall prediction of the model.

When interpreting the predictions of one sample, the SHAP method considers the combination of all features and calculates the average contribution of each feature value to the model predictions. The SHAP value is used to measure the contribution of each feature to the model’s prediction outcome. The average SHAP value usually refers to the value calculated by averaging the SHAP value of a feature in multiple samples, and by calculating the average SHAP value of each feature value, we can obtain the average influence of the feature on the model prediction. The wavelengths of the top 50 SHAP values for the PLSR and CNN models can be found in Table S1 in the Supplementary Materials file. The results show that there is a certain degree of similarity in the selection of characteristic wavelengths between PLSR models under different varieties, while there are obvious differences in the important wavelengths selected by CNN models. Specifically, for Ponkan, there is significant overlapping in the characteristic wavelengths of the PLSR and CNN models, indicating that the key information extracted by the two methods on the variety is relatively consistent. In contrast, for Tianchao, the wavelengths selected by the PLSR and the CNN model coincide less, indicating that there are great differences in the spectral features of different modeling methods on this variety.

**Figure 4:**
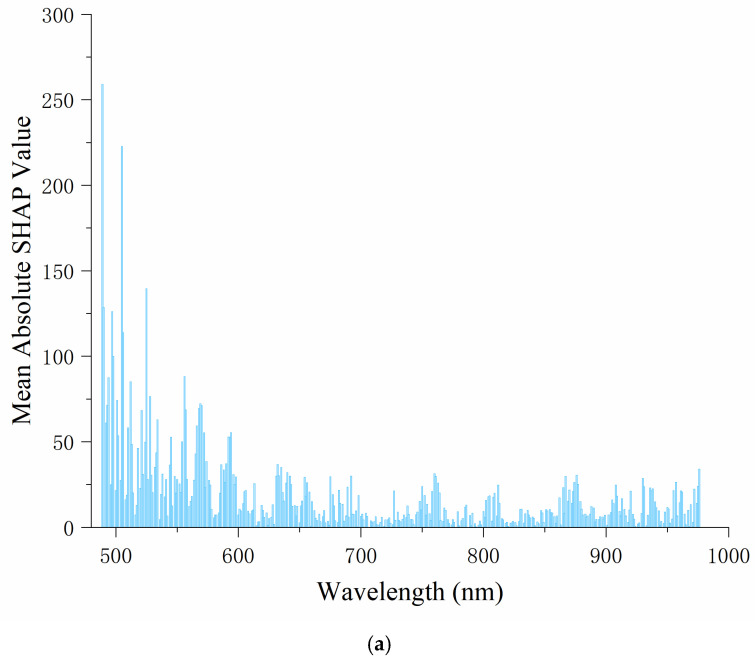

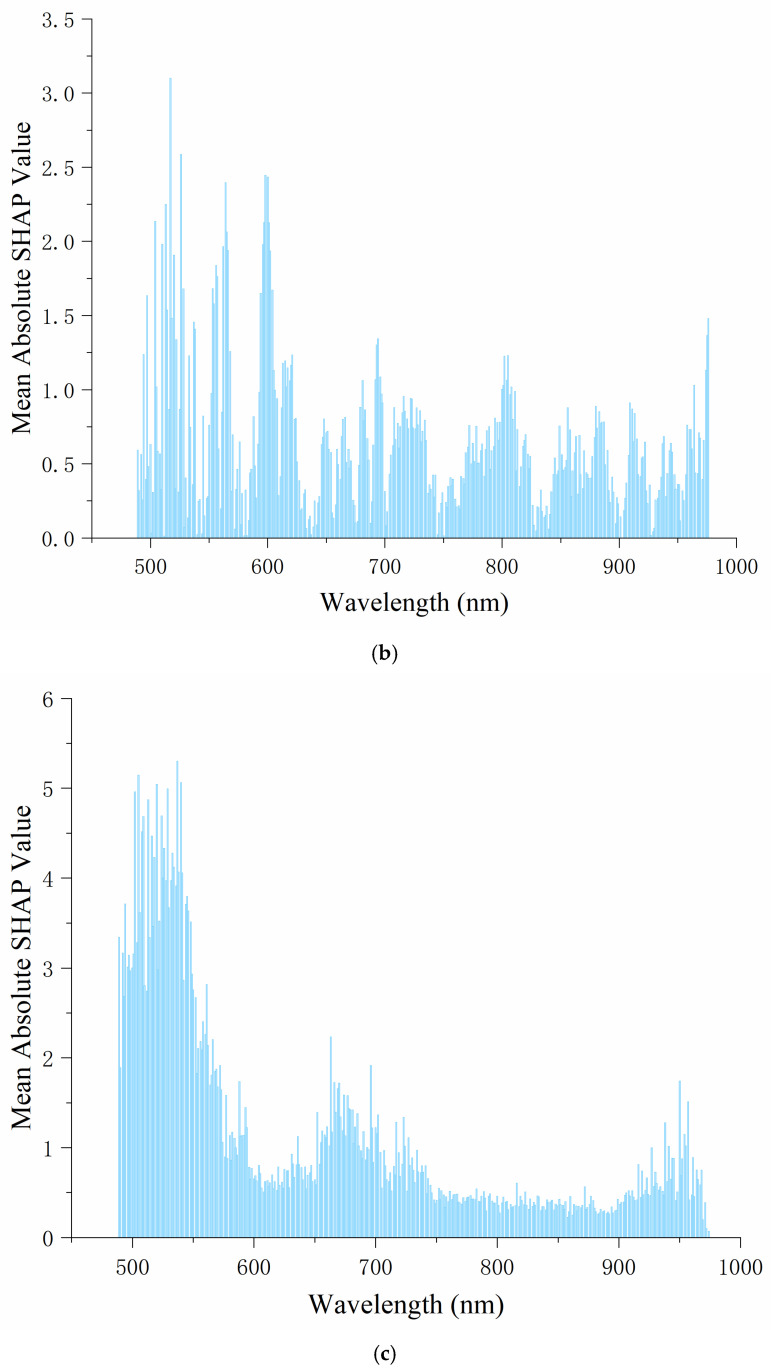

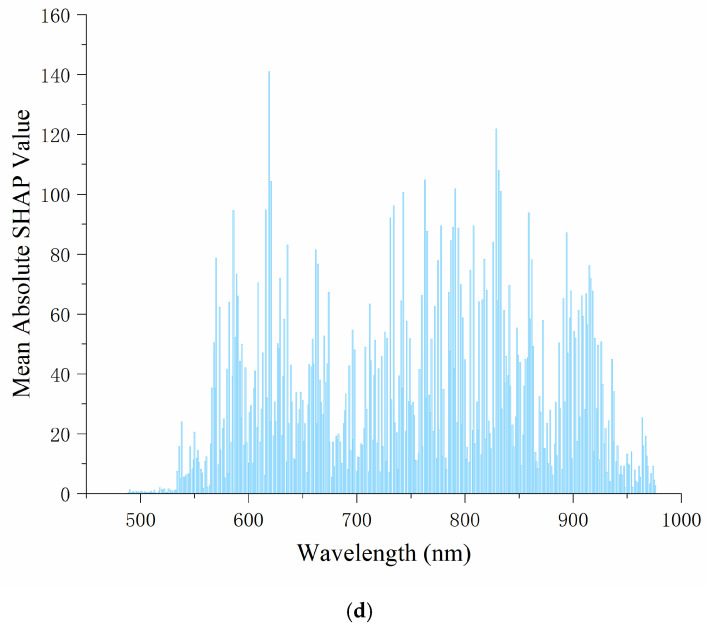
Explanation of the PLSR and CNN by SHAP visualization: (**a**) the PLSR model of Ponkan; (**b**) the PLSR model of Tianchao; (**c**) the CNN model of Ponkan; (**d**) the CNN model of Tianchao.

In Figure 4, the horizontal axis represents the wavelength, and the vertical axis represents the average absolute SHAP value, which reflects the contribution of the wavelength to the model’s prediction results. The higher the value, the more important the wavelength is in the model. It can be seen in Figure 4 that in both the PLSR and CNN models, the feature importance distribution of Ponkan is relatively stable, with higher average absolute SHAP values around 500–600 nm, and then gradually decreasing. However, the average SHAP value distribution of Tianchao is more scattered, without a clear overall decreasing or increasing trend. Overall, for Ponkan, the wavelength influence is concentrated in a specific range, and the degree is relatively balanced, which may be because the spectral information in this band is closely related to the intrinsic quality and appearance features of this variety of oranges. For Tianchao, the wavelength influence is more dispersed, and some wavelengths have a very high influence degree. The model does not overly rely on a single wavelength range, and the features of different wavelengths work together to affect the prediction. This may be because the relevant features of this variety of oranges are reflected in a wider spectral range. These differences may stem from the differences in the composition and structure of different varieties of citrus, which cause the importance of spectral information at different wavelengths to vary under the same model.

## 4. Discussion

In this study, advanced machine learning and deep learning techniques for building a predictive model based on the HSI dataset to accurately estimate the SSC of different citrus varieties (Ponkan and Tianchao) were explored. The experiments of the two varieties were conducted under the same conditions, and the differences in the model performances for SSC prediction of the two varieties of citrus fruits could be observed. The reason might be the differences in intrinsic physicochemical properties of the different varieties of citrus fruits, such as tissue structure and light absorbance, reflectance, and scattering. Another possible reason would be the differences in the sample distribution of the two varieties of citrus fruits.

Although deep learning models are known for their powerful automatic feature extraction capabilities and can capture complex data patterns, they have not overwhelmed traditional machine learning models in this practice. The reasons are discussed in detail as follows.

Traditional machine learning models, such as PLSR, have demonstrated efficient fitting and generalization performance in some specific sample distributions with their concise structures and mature theoretical foundations. In computer programming, PLSR uses direct matrix operations to calculate the weights and biases of the linear model. The range and magnitude of input and output values will not affect modeling. However, CNN models cannot use such methods for their weights and biases calculation due to their complex multi-layer architectures. An iterative method is always used to adjust those parameters. In each epoch of the iterative loop, the change of all parameters depends on the calculated gradient (direction) and a fixed learning rate (magnitude). It must be emphasized that the weight values *k*_1_ to *k_n_* and bias *b* might be on different scales. The model of a single neuron is given as follows:

(2) $$y=\emptyset (k_{1}x_{1}+k_{2}x_{2}+\ldots +k_{n}x_{n}+b)$$

where ∅ is the activation function, *y* is the output of the neuron. Suppose the expected *k*_1_ is at 10^−3^ level, while the expected *b* is about 10^3^, it is very hard to find a fixed learning rate to tune all these parameters. Since the processed data in this study was the spectrum of fruits (a vector composed of 358 elements), the selection of a suitable learning rate and model optimization might be more challenging. On the contrary, traditional machine learning models could better handle the simple and linear-pattern data. Therefore, in some studies, deep learning methods have shown relatively better performance over the traditional machine learning methods used in some other studies [15,16,17,29,30]. However, in some other studies, the traditional machine learning methods performed better [13,14].

On the other hand, the number of samples and the pattern of the data might also affect the performance of the deep learning models used. For example, in hyperspectral imaging-based crop seed classification tasks, deep learning reached relatively equal performances to simple traditional models when processing small-scale datasets [31], while deep learning models showed significant superiority in processing relatively large and complex datasets [32]. The success of traditional machine learning algorithms has been proven based on decades of research [7,12,13]. However, deep learning models have shown good potential in extracting complex feature relationships, but in the prediction tasks with limited data volume or relatively simple feature rules, they have been prone to overfitting, and the model performance fluctuates greatly [33]. Hence, in engineering applications, the modeling method should be selected and optimized based on actual situations.

The SHAP-based wavelength importance analysis revealed notable differences in the selected spectral features between PLSR and CNN models, particularly across different citrus varieties. For the Ponkan, a high degree of overlap was observed between the top 50 wavelengths identified by both PLSR and CNN, indicating that the key predictive features were largely consistent regardless of the modeling approach. This suggests that the underlying spectral information relevant to the target trait in Ponkan is linearly correlated and robust across model types. In contrast, the Tianchao exhibited minimal overlap in selected wavelengths between the two models. This divergence may be attributed to the differences in modeling mechanisms; PLSR relies on linear latent variable projection, thus favoring features with strong global linear relationships to the output, while CNN captures localized and nonlinear interactions through convolutional layers. The reduced agreement in Tianchao implies a more complex or nonlinear relationship between spectral features and the prediction target, which CNN is more capable of uncovering. These observations highlight the influence of both model architecture and data-specific spectral characteristics on feature importance, emphasizing the need to tailor wavelength selection strategies to the nature of the data and the modeling task.

In this study, only the *r* of the training, validation, and test sets obtained by the PLSR model for Ponkan were all over 0.8. The overall prediction performances of SSC by different models in this research were slightly worse than those in the research [8]. However, the prediction performance of SSC in this study was much worse than that in the research [34,35]. These differences might be attributed to various reasons, such as different experimental conditions and operations, different citrus varieties, different sampling strategies, different imaging instruments and strategies, different data analysis strategies, and so on. Moreover, the impact of varietal differences on the prediction performance of SSC could be observed in this study. In some other studies using VNIR spectra for citrus SSC prediction, the varietal differences could also be observed [3,36,37].

Although promising results were obtained in this study, there were several limitations that should be addressed. In the past, new modeling methods have received extensive research and attention. At the current stage, researchers have realized the importance of high-quality datasets. A dataset of large sample size, covering various sampling zones and cultivars used, might better leverage the role of complex deep learning models to achieve a breakthrough in detection accuracy. In this study, the variety and source of the studied samples are relatively limited. Moreover, the concept of standardized data collection needs to be given attention. By standardizing the data collection methods, the arrangement of collection points on fruits, and the requirements for collection of environmental parameters, it is possible to make the data collected by different researchers tend towards a similar pattern, which helps to make the models built by different researchers interchangeable. Besides, it should also be noted that the performances of deep learning models were determined by many factors, including the model structures, parameters, and hyperparameters. Exploring the optimal model structures manually was challenging. It is suggested that the method of applying different learning rates to weights and bias at different levels should be explored. Some advanced technologies, such as larger model-assisted optimization, could also be studied. For example, the popular GPT-4 was used for optimizing a YOLO detection model [38].

In summary, this study systematically compared the performance of different modeling strategies in the prediction of SSC of multi-variety citrus, verifying the potential of hyperspectral imaging with machine learning and deep learning algorithms. However, there were still great challenges in the construction of citrus SSC prediction models to cope with the variety and diversity, and to balance the complexity and performance of the models. Given the good performance of traditional machine learning algorithms and deep learning algorithms for SSC prediction, future works can focus on dealing with the varietal differences to improve the prediction performance of different varieties. More efforts should be made to explore the application scenes of deep learning and traditional machine learning methods, and to further investigate when, where, and how to use deep learning and traditional machine learning methods. Deep insights should be explored for the data structure and feature distribution of spectral data on deep learning performances, helping to explore the optimal deep learning model architectures. More efforts should also be undertaken to achieve model generalization ability enhancement and cross-variety adaptability optimization with more samples and more varieties, to promote the practical application of hyperspectral technology in rapid fruit quality detection.

## 5. Conclusions

In this study, the application of hyperspectral imaging, machine learning, and deep learning in the determination of SSC in citrus fruits was successfully explored. The differences in model performances for different citrus varieties were investigated. The machine learning and deep learning models were constructed and compared. There were certain differences in spectral characteristics between different varieties, which led to different degrees of performance fluctuations in the SSC prediction of different varieties. Machine learning models showed generally equivalent or better performance than deep learning models (including the combined deep learning models). Through SHAP-based ranking of characteristic wavelengths in PLSR and CNN models, it was observed that the Ponkan variety showed a high degree of overlap in selected wavelengths across both models, indicating more stable spectral features and greater consistency between linear and nonlinear approaches. In contrast, the Tianchao variety exhibited significant differences in wavelength selection, suggesting that its spectral characteristics are more model-dependent. This phenomenon highlights the varietal differences in spectral feature representation and underscores the importance of selecting appropriate modeling strategies that align with the linearity or nonlinearity of the data. Optimizing model architecture not only improves predictive performance but also enhances the interpretability of key wavelengths, which is crucial for developing reliable and explainable spectral analysis systems. Future research should aim to enhance the prediction performance across different citrus varieties and further investigate strategies for applying machine learning and deep learning to spectral data analysis. This includes exploring how to effectively harness the respective strengths of these modeling approaches. In addition, the development of advanced deep learning architectures should be explored to fully realize their potential in hyperspectral analysis. Expanding the sample size in future studies is also essential for building more robust and accurate predictive models.

## Figures and Tables

**Table 1:** Statistical analysis of SSC for different varieties of citrus fruit.

	Training Set (%)			Validation Set (%)			Testing Set (%)		
	Number	Min	Max	Number	Min	Max	Number	Min	Max
Ponkan	184	10.3	15.8	46	10.4	15.3	46	10.8	14.7
Tianchao	180	8.8	14.1	44	7.6	13.1	44	8.1	13.1

**Table 2:** Traditional machine learning models’ evaluation results for SSC.

		Training Set		Validation Set		Testing Set	
		*r_c_*	RMSEC	*r_v_*	RMSEV	*r_p_*	RMSEP
**PLSR**	Ponkan	0.899	0.432	0.845	0.591	0.820	0.562
	Tianchao	0.836	0.582	0.796	0.678	0.749	0.728
**SVR**	Ponkan	0.782	0.623	0.743	0.689	0.719	0.769
	Tianchao	0.794	0.643	0.732	0.768	0.712	0.831

**Table 3:** Deep learning models evaluation results for SSC.

		Training Set		Validation Set		Testing Set	
		*r_c_*	RMSEC	*r_v_*	RMSEV	*r_p_*	RMSEP
**CNN**	Ponkan	0.742	1.413	0.706	1.477	0.687	1.432
	Tianchao	0.846	0.612	0.760	0.744	0.754	0.746
**LSTM**	Ponkan	0.718	0.691	0.659	0.755	0.653	0.832
	Tianchao	0.687	0.858	0.619	1.009	0.613	1.024
**Transformer**	Ponkan	0.713	0.759	0.703	0.760	0.650	0.869
	Tianchao	0.713	0.795	0.656	0.972	0.641	0.905
**CNN–LSTM**	Ponkan	0.806	0.5934	0.723	0.669	0.723	0.773
	Tianchao	0.793	0.724	0.734	0.780	0.709	0.843
**LSTM–CNN**	Ponkan	0.749	0.667	0.742	0.791	0.717	0.694
	Tianchao	0.759	0.810	0.701	0.878	0.690	0.832
**CNN–Transformer**	Ponkan	0.754	0.773	0.676	0.821	0.654	0.943
	Tianchao	0.748	1.567	0.660	1.574	0.648	0.726
**Transformer–CNN**	Ponkan	0.738	0.672	0.692	0.709	0.690	0.804
	Tianchao	0.746	1.021	0.659	1.251	0.646	1.265

## Data Availability

The original contributions presented in this study are included in the article/Supplementary Materials. Further inquiries can be directed to the corresponding author.

## Supplementary Materials

The following supporting information can be downloaded at: https://www.mdpi.com/article/10.3390/foods14122091/s1, Table S1: Top 50 SHAP-Based wavelengths in PLSR and CNN models for Ponkan and Tianchao mandarins. Figure S1: RGB images of the four sampling sides of a citrus fruit of Tiancao. Figure S2: RGB images of the four sampling sides of a citrus fruit of Ponkan. Figure S3: The structure diagram of CNN model: (a) Ponkan mandarin; (b) Tianchao mandarin. Figure S4: The structure diagram of LSTM model: (a) Ponkan mandarin;(b) Tianchao mandarin. Figure S5: The structure diagram of CNN-LSTM model and LSTM-CNN model: (a) CNN-LSTM of Ponkan mandarin; (b) CNN-LSTM of Tianchao mandarin; (c) LSTM-CNN of Ponkan mandarin; (d) LSTM-CNN of Tianchao mandarin. Figure S6: The structure diagram of Transformer model: (a) Ponkan mandarin; (b) Tianchao mandarin. Figure S7: The structure diagram of CNN-Transformer model and Transformer-CNN model: (a) CNN-Transformer of Ponkan mandarin;(b) CNN-Transformer of Tianchao mandarin; (c) Transformer-CNN of Ponkan mandarin; (d) Transformer-CNN of Tianchao mandarin.
