# Supplementary material for: Detection of Soluble Solid Content in Citrus Fruits Using Hyperspectral Imaging with Machine and Deep Learning: A Comparative Study of Two Citrus Cultivars
Source: Foods. 2025 Jun 13;14(12):2091. doi: 10.3390/foods14122091 (PMC12192198; doi:10.3390/foods14122091)
Supplement: Supplementary file 1 [file foods-14-02091-s001.zip › foods-3659735-supplementary.pdf]

Supplementary materials

Table S1. Top 50 SHAP-Based wavelengths in PLSR and CNN models for Ponkan and Tianchao mandarins.

PG		TC	
**PLSR**	**CNN**	**PLSR**	**CNN**
**489**	537	517	**619**
**505**	**505**	526	829
**525**	540	598	831
490	520	600	763
**497**	**529**	564	**621**
**506**	**502**	513	791
**498**	**513**	504	833
556	**524**	597	743
**494**	509	601	734
**512**	508	565	616
**528**	516	510	586
**501**	526	596	859
569	**533**	562	731
**493**	**518**	566	778
570	**534**	602	808
568	**538**	520	789
557	541	556	794
**521**	**525**	557	765
**534**	**532**	553	894
**492**	**528**	528	787
566	536	604	826
**510**	**545**	594	636
594	**494**	497	662
572	**544**	554	570
**502**	530	514	818
592	546	518	862
**545**	**506**	976	775
593	**522**	537	664
554	548	538	915
**524**	517	975	**805**
**513**	**489**	694	589
**518**	514	522	629
**533**	504	693	916
565	**492**	568	609
574	**501**	494	796
590	**497**	**621**	841

632	496	**805**	820
586	500	533	918
**544**	**521**	802	898
635	**498**	615	785
**532**	549	613	674
976	542	620	912
588	561	617	760
640	**510**	974	908
760	550	605	590
**538**	**512**	696	891
**522**	**493**	692	816
596	552	804	830
**529**	558	681	741
876	560	**619**	813

[#] The PLSR of Ponkan and the same values in the top 50 wavelengths of CNN are marked in bold red; The PLSR of Tianchao and the same values in the top 50 wavelengths of CNN are marked in bold blue.

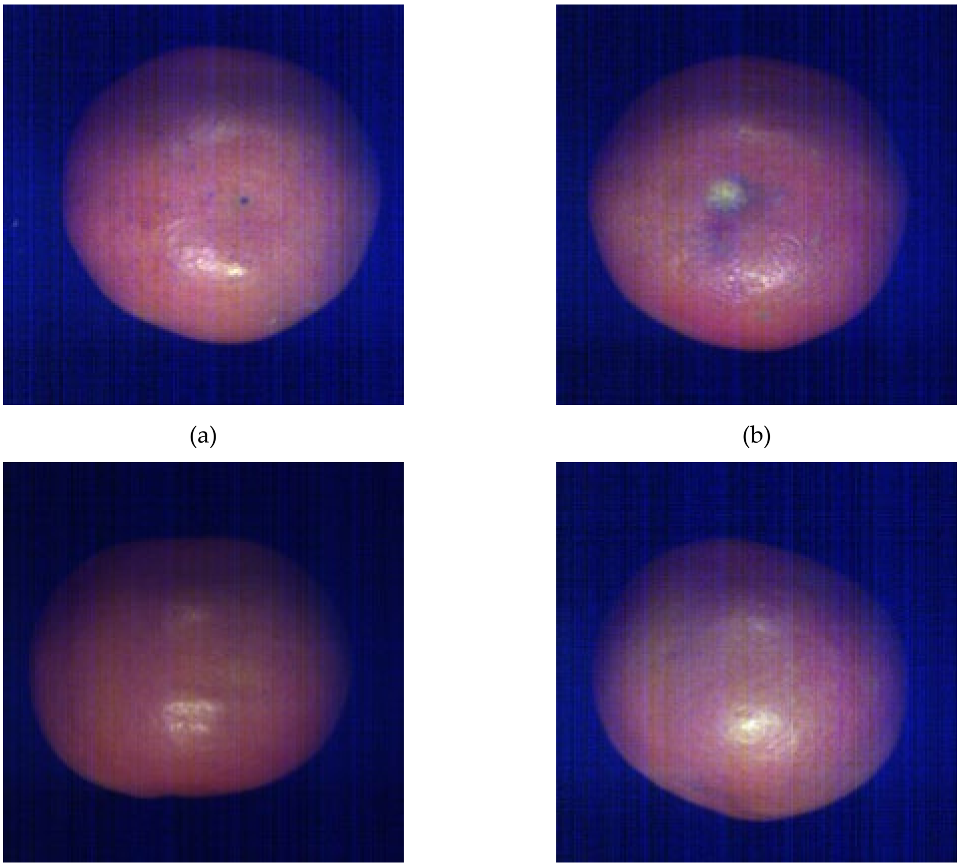

(a) (b) (c) (d)

Figure S1. RGB images of the four sampling sides of a citrus fruit of Tiancao.

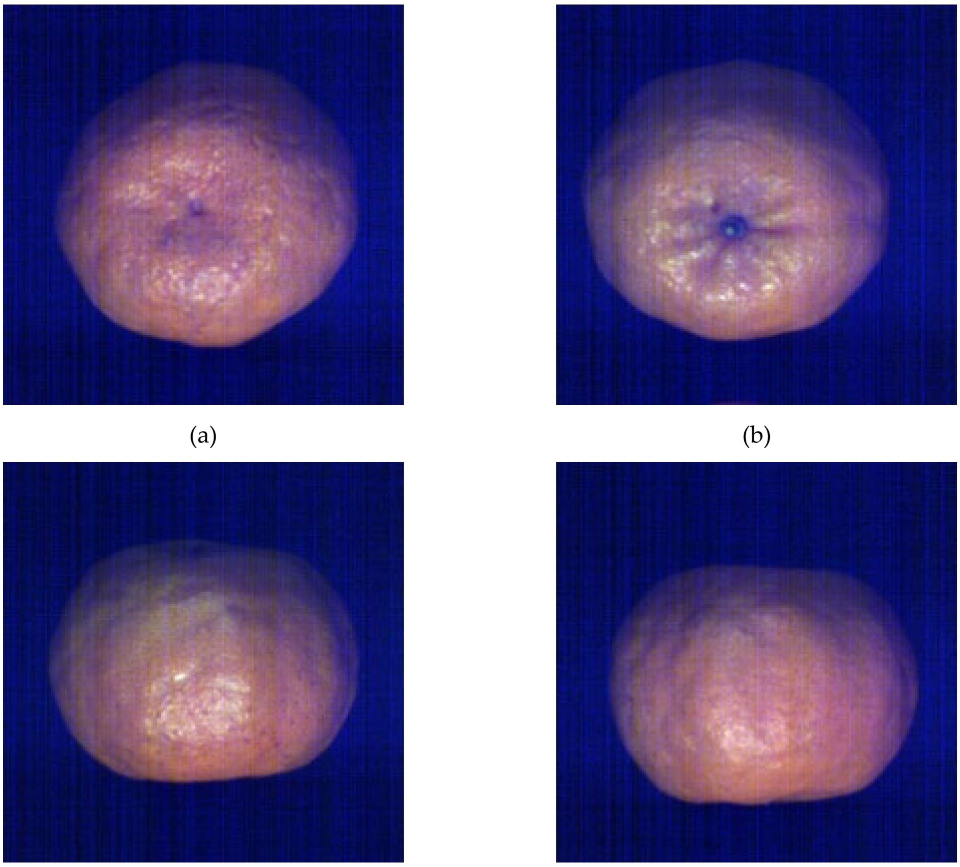

(a) (b)

(c) (d)

Figure S2. RGB images of the four sampling sides of a citrus fruit of Ponkan.

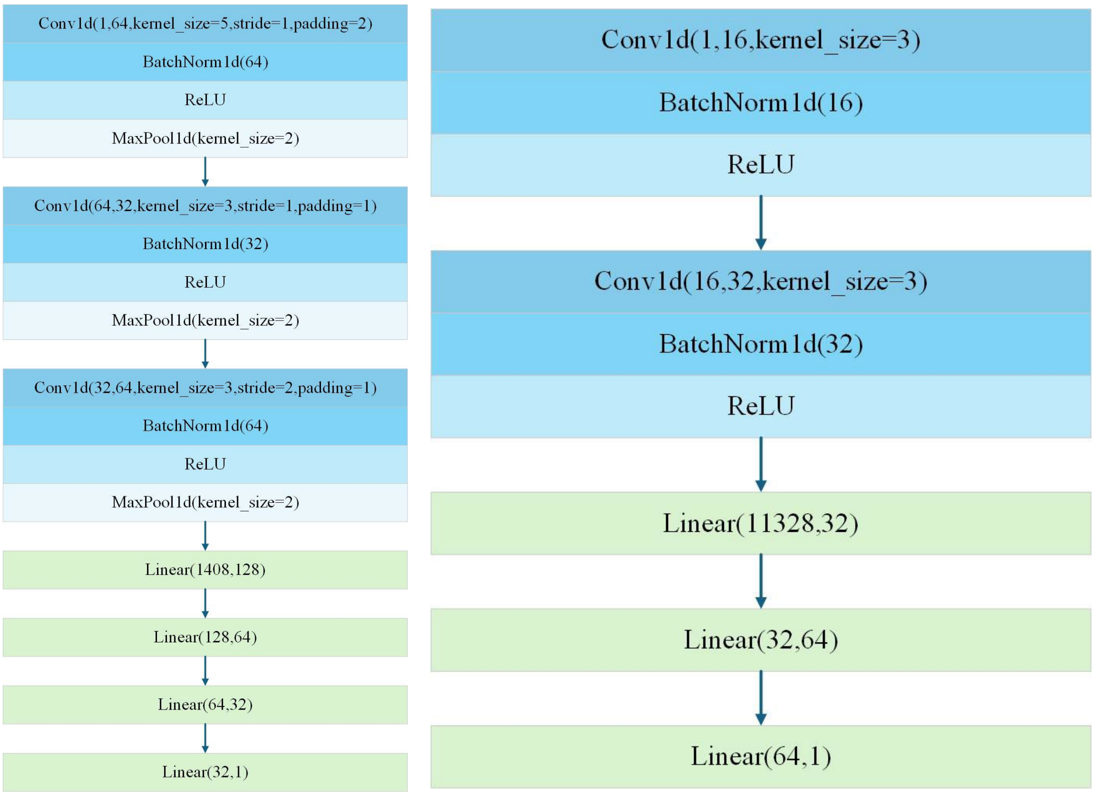

(a) (b)

Figure S3. The structure diagram of CNN model: (a) Ponkan mandarin; (b) Tianchao mandarin.

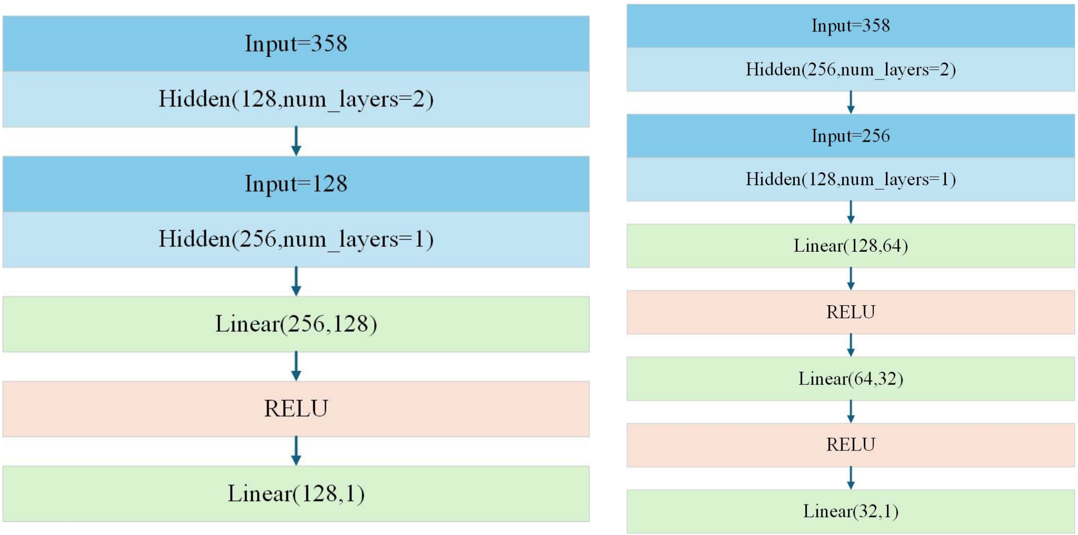

(a) (b)

Figure S4. The structure diagram of LSTM model: (a) Ponkan mandarin; (b) Tianchao mandarin.

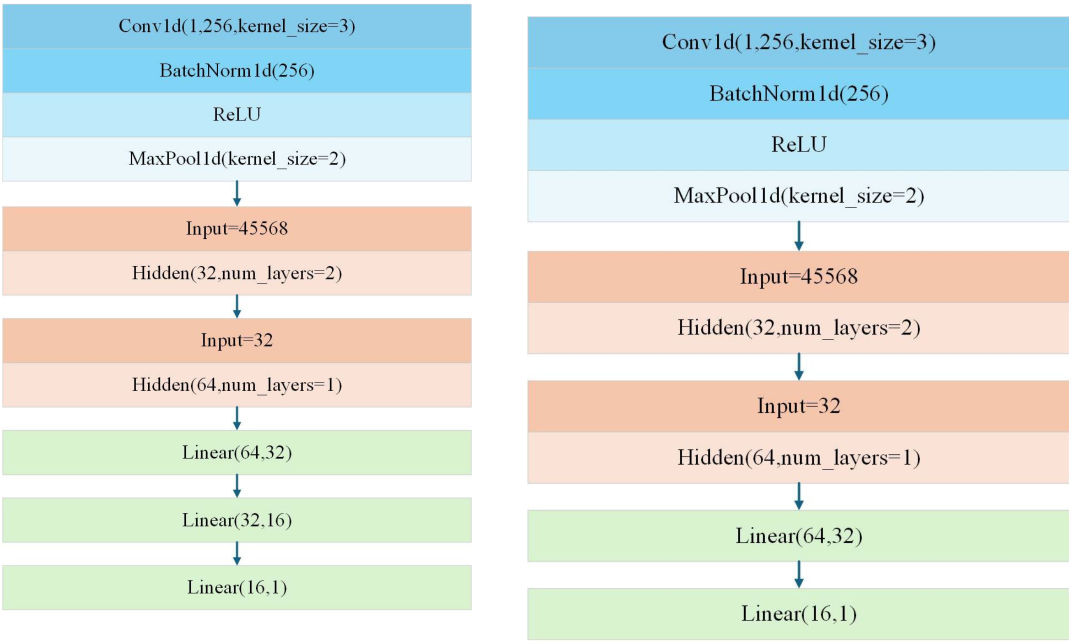

(a) (b)

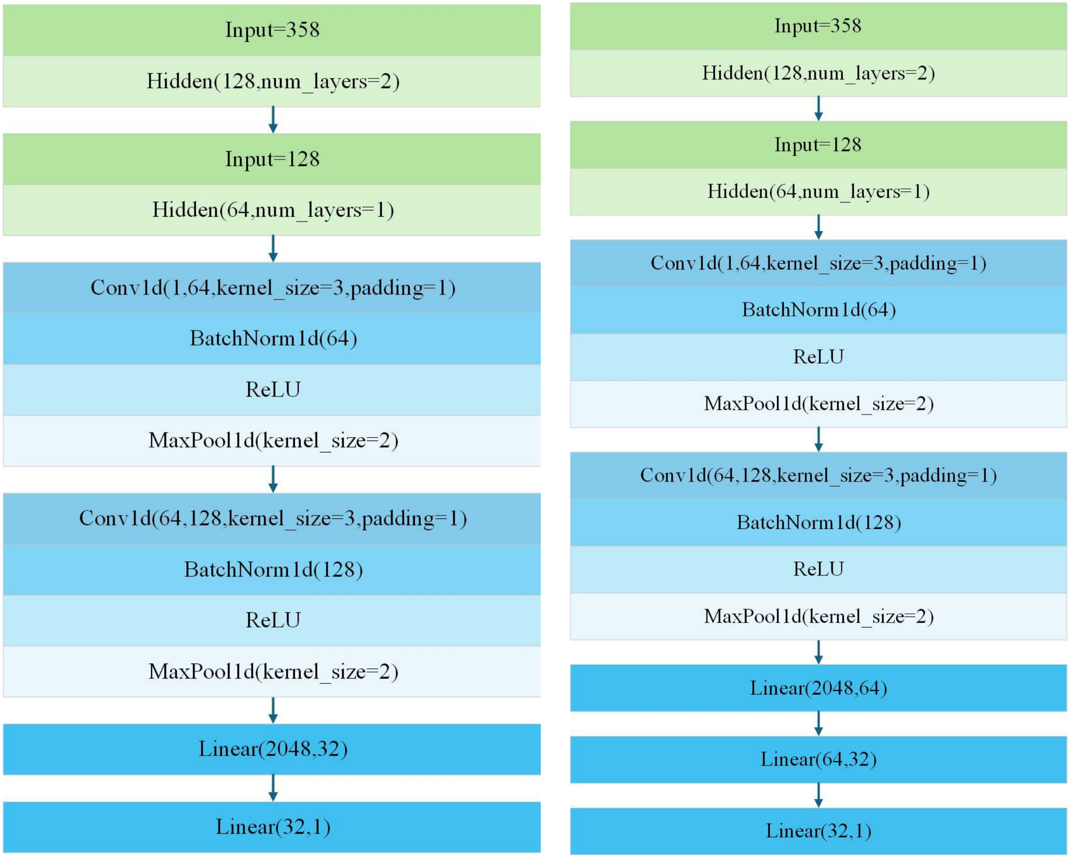

(c) (d)

Figure S5. The structure diagram of CNN-LSTM model and LSTM-CNN model: (a) CNN-LSTM of Ponkan mandarin; (b) CNN-LSTM of Tianchao mandarin; (c) LSTM-CNN of Ponkan mandarin; (d) LSTM-CNN of Tianchao mandarin.

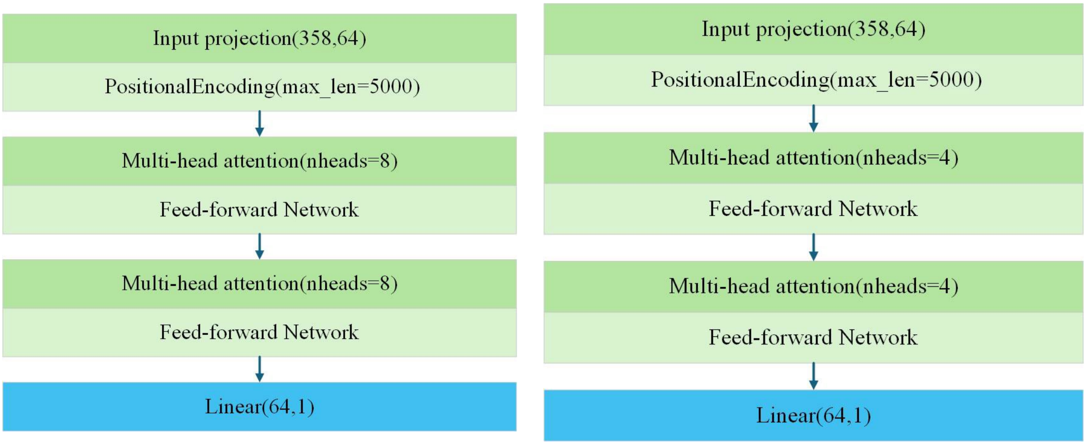

(a) (b)

Figure S6. The structure diagram of Transformer model: (a) Ponkan mandarin; (b) Tianchao mandarin.

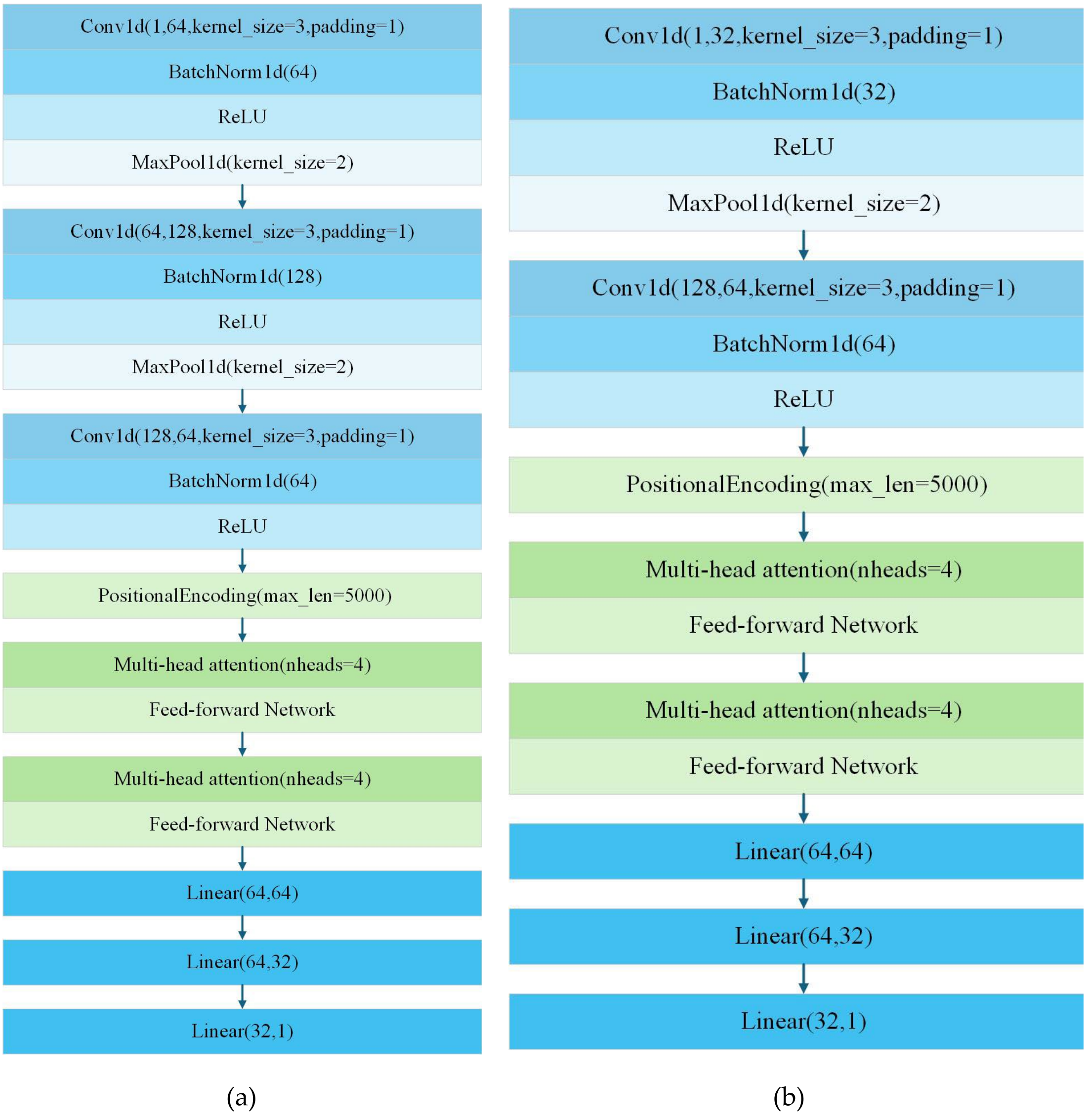
Conv1d(1,64,kernel_size=3,padding=1)
BatchNorm1d(64)
ReLU
MaxPool1d(kernel_size=2)
Conv1d(64,128,kernel_size=3,padding=1)
BatchNorm1d(128)
ReLU
MaxPool1d(kernel_size=2)
Conv1d(128,64,kernel_size=3,padding=1)
BatchNorm1d(64)
ReLU
PositionalEncoding(max_len=5000)
Multi-head attention(nheads=4)
Feed-forward Network
Multi-head attention(nheads=4)
Feed-forward Network
Linear(64,64)
Linear(64,32)
Linear(32,1)
(a)
Conv1d(1,32,kernel_size=3,padding=1)
BatchNorm1d(32)
ReLU
MaxPool1d(kernel_size=2)
Conv1d(128,64,kernel_size=3,padding=1)
BatchNorm1d(64)
ReLU
PositionalEncoding(max_len=5000)
Multi-head attention(nheads=4)
Feed-forward Network
Multi-head attention(nheads=4)
Feed-forward Network
Linear(64,64)
Linear(64,32)
Linear(32,1)
(b)

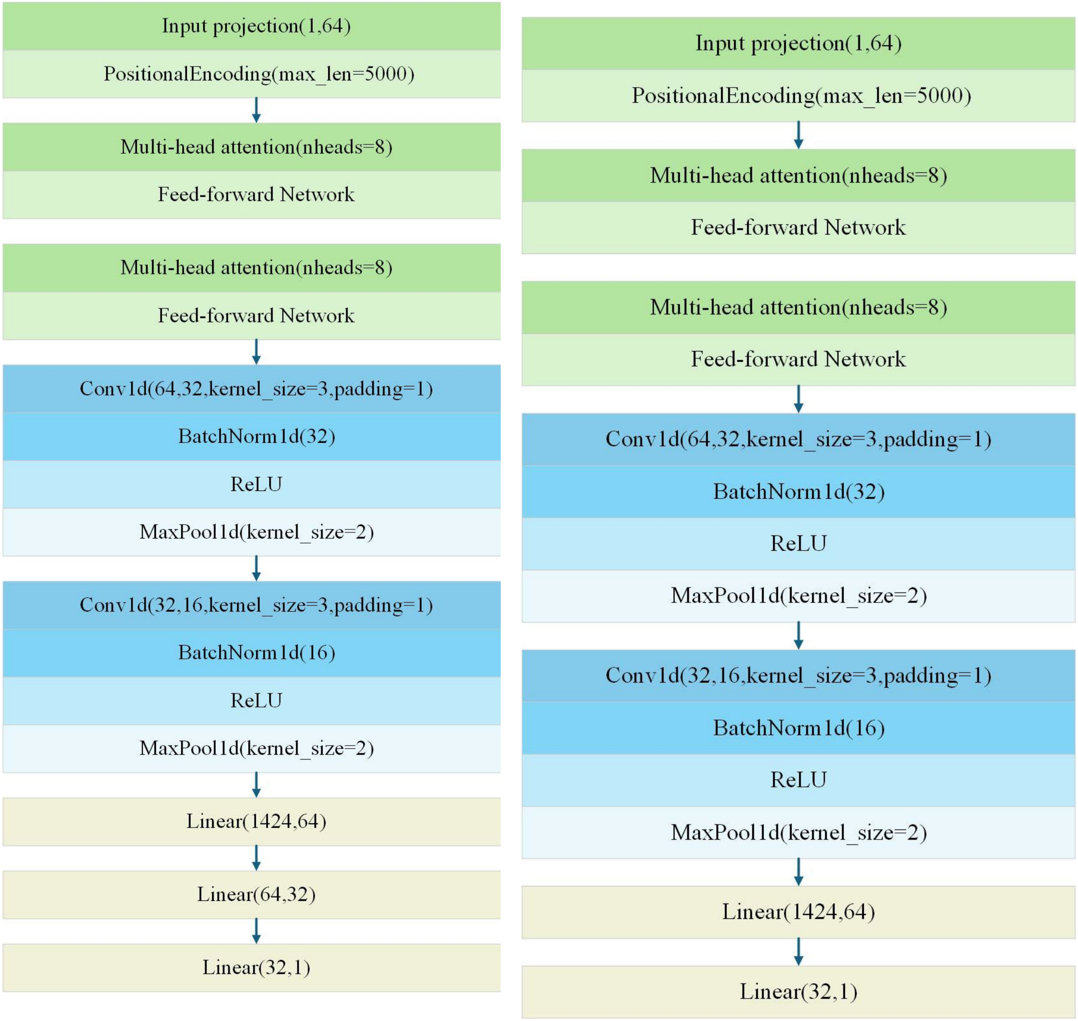

(c) (d)

Figure S7. The structure diagram of CNN-Transformer model and Transformer-CNN model: (a) CNN-Transformer of Ponkan mandarin;(b) CNN-Transformer of Tianchao mandarin; (c) Transformer-CNN of Ponkan mandarin; (d) Transformer-CNN of Tianchao mandarin.
